# Small-area distribution of multiple sclerosis incidence in western France: in search of environmental triggers

**DOI:** 10.1186/s12942-017-0108-6

**Published:** 2017-09-21

**Authors:** Karima Hammas, Jacqueline Yaouanq, Morgane Lannes, Gilles Edan, Jean-François Viel

**Affiliations:** 1 Department of Epidemiology and Public Health, University Hospital, 2 rue Henri Le Guilloux, 35033 Rennes, France; 2 Department of Neurology, University Hospital, Rennes, France; 3 INSERM-IRSET No 1085, Epidemiological Research on Environment, Reproduction and Development, University of Rennes 1, Rennes, France

**Keywords:** Multiple sclerosis, Small-area distribution, Spatial cluster, Environmental triggers

## Abstract

**Background:**

Despite intensive research over several decades, the etiology of multiple sclerosis (MS) remains poorly understood, although environmental factors are supposedly implicated. Our goal was to identify spatial clusters of MS incident cases at the small-area level to provide clues to local environmental risk factors that might cause or trigger the disease.

**Methods:**

A population-based and multi-stage study was performed in the French Brittany region to accurately ascertain the clinical onset of disease during the 2000–2004 period. The municipality of residence at the time of clinical onset was geocoded. To test for the presence of MS incidence clusters and to identify their approximate locations, we used a spatial scan statistic. We adjusted for socioeconomic deprivation, known to be strongly associated with increased MS incident rates, and scanned simultaneously for areas with either high or low rates. Sensitivity analyses (focusing on relapsing-remitting forms and/or places of residence available within the year following clinical onset) were performed.

**Results:**

A total of 848 incident cases of MS were registered in Brittany, corresponding to a crude annual incidence rate of 5.8 per 100,000 inhabitants. The spatial scan statistic did not find a significant cluster of MS incidence in either the primary analysis (*p* value ≥ 0.56) or in the sensitivity analyses (*p* value ≥ 0.16).

**Conclusion:**

The findings of this study indicate that MS incidence is not markedly affected across space, suggesting that in the years preceding the first clinical expression of the disease, no environmental trigger is operative at the small-area population level in the French Brittany region.

## Background

Multiple sclerosis (MS) is a chronic immune-mediated disease of the central nervous system characterized by chronic inflammation, demyelination, gliosis, and axonal pathology. Despite intensive research over several decades and early identification of susceptibility genes, the etiology of MS remains poorly understood, although environmental factors are supposedly implicated. A number of factors—birth-cohort and season-of-birth effects, changing incidence among populations of common ancestry migrating to environments with different risks, latitude-dependent incidence and a high discordance rate among monozygotic twins—indicate that MS is a complex multifactorial disease. It is now generally agreed that MS susceptibility is dependent on the elaborate interaction of a variety of low-penetrance genetic factors and environmental agents that, by themselves, convey low risk, but when combined result in disease initiation [1–4].

According to Ebers [5], re-examination of the conceptual and practical importance of the environment in the pathogenesis of MS seems timely, and in this respect, the uneven geographical distribution of MS is central to understanding the role of environment.

Looking beyond the well-established large geographic variations [5], localized clustering of MS incident cases may provide clues to environmental risk factors that might cause or trigger the disease. Indeed, cluster analysis allows researchers to assess whether people with MS have lived closer to one another than would be expected by chance during a specific time period, in which case they would have been exposed to a common environmental risk factor. So far, there are few documented examples of MS clusters at the small-area level [6, 7].

France is considered a country of medium-to-high MS prevalence and incidence. Recent studies have shown marked geographical disparities in MS incidence with higher rates in north-eastern France (and lower rates in the south-east), but this conclusion was reached using a single source of administrative data from the main French health insurance system (collected for health system management rather than research purposes), and the study was conducted at a large geographical level (“départements” averaging 625,000 inhabitants) [8, 9].

The goal of this study was, therefore, to identify spatial clusters of MS incident cases in the populations of small geographic areas to suggest novel environmental hypotheses for the disease.

## Methods

### Study area

The Brittany region is the largest peninsula of France. Located in the northwest, it covers 27,209 km^2^ (5% of France) and has a temperate oceanic climate (mean annual temperature of 11.2 °C). In 2013, its population was 3,258,707 inhabitants (1,583,540 men and 1,675,167 women) residing in 1270 municipalities.

### Case ascertainment

To be eligible, patients had to be residing in Brittany when they first experienced MS symptoms, and their symptoms had to occur between 1 January 2000 and 31 December 2004.

Incidence was based on clinical onset, the timing of which may be difficult to establish given the complex course of symptom appearance and process of diagnosis. Indeed, when first visiting a neurologist, patients can present a second event with historical evidence of one past attack consistent with demyelination, or, for insidious neurological progression, suggestive of primary progressive MS. Moreover, even if they present a first neurologic episode of inflammation or demyelination (called clinically isolated syndrome, CIS) [10, 11], making a definite diagnosis of MS can be a lengthy process because a second event (neurologic examination, visual evoked potential response in patients with prior visual disturbance, or MRI consistent with demyelination in the central nervous system region) can occur years after the prior neurologic symptom.

To tackle this epidemiological challenge, MS has been identified using patient records at hospitals and at private neurological practices that provide on-going care to the Brittany population (including the University hospital in the neighboring Pays-de-Loire region, which could evaluate patients seeking medical attention outside their zone of residence). Data files were available from a previous epidemiological study conducted during 2000–2004, which assessed MS incidence during 2000–2001 [12]. We extended this serial follow-up to 2016 to correct the effects of diagnostic delays. MS diagnosis could, therefore, be made during the 2005–2016 time period but allocated to the year of the first symptom (onset of disease) if the latter occurred during the 2000–2004 time period. Moreover, we used an additional source of case identification. Minimum inpatient datasets, including the International Classification of Diseases-10 code related to MS (G35), were acquired from hospital medical record departments of the region for the same time period. Patients initially diagnosed with MS who later received another diagnosis were excluded from the study.

A further difficulty was that the diagnostic criteria of Poser were used by neurologists at the beginning of the study period but were progressively superseded by the McDonald system. Therefore, following Yaouanq et al [12], to ultimately be included in the study, incident cases had to fulfill the McDonald criteria for MS or at least the Poser criteria for probable or definite MS. All patient charts were reviewed and scrutinized by only one neurologist (JY) for data on gender, year of birth, course of disease at onset, year of disease onset, year of first clinical examination for symptoms suggestive of MS, and place of residence at time of clinical onset. In cases where the location at clinical onset or the location within the following year (120 of 848, 14%) was missing, the nearest place of residence available from medical records was used.

### Population data

Population counts by municipality, gender, and 5-year age groups were obtained from the French Office of Population Censuses for the year 1999. Because population estimates were not available for intercensal years, the 1999 population counts were multiplied by 5 to estimate the population at risk for the 5-year period 2000–2004.

### Statistical methods

The region wide MS incidence rate per 100,000 persons was age-standardized to the standard European population (2013) by means of the direct method.

To test for the presence of disease clusters and to identify their approximate locations, we used the spatial scan statistic developed by Kulldorff et al. [13–15], and scanned simultaneously for areas with either high or low rates. The municipality of residence at the time of clinical onset was geocoded. We assumed the observed number of incident cases from a municipality to be Poisson distributed. The expected numbers of MS cases for each municipality were computed by applying an internal standard (i.e., incidence rates from the entire region for the same years) to the person-years of each area and stratifying by gender, 5-year age categories and socioeconomic deprivation (known to be strongly associated with increased MS incident rates [6, 7]). Socioeconomic deprivation was assessed using a French deprivation index, an area-based measure of the disadvantage experienced by a neighborhood [16]. A circular window moved from the centroid of one municipality to the centroid of another municipality. For each location, the radius varied continuously from zero to a maximum, such that—at most—30% of the region’s population was included (when the window included a centroid, the population related to the municipality was included as a whole in the window). For each location and size of the scanning window, the null hypothesis was that the incidence rate was the same for each municipality. Conversely, under the alternative hypothesis, the incidence rate was assumed to be lower or higher inside than outside the window. During the spatial process, an enormous number of different circles, including different sets of neighboring municipalities, were evaluated to find the most likely cluster. A likelihood function was calculated for each circle and was maximized over all of them, identifying the zone that represented the most likely MS cluster (i.e., the cluster that is least likely to have occurred by chance). Secondary clusters in the data set that did not overlap spatially with the most likely cluster were also reported.

For hypothesis testing, we used a Monte Carlo procedure to generate 9999 random replications of the data set under the null hypothesis (scanning over the possible locations and sizes). The maximum likelihood ratio test statistic was calculated for each random replication as well as for the real data set; if the latter was among the 5% highest, then the test was significant at the 0.05 level.

Three sensitivity analyses were conducted to assess the robustness of the findings from the primary analysis (whole dataset) and to ensure their appropriate interpretation. First, only relapsing-remitting (RRMS) forms were considered (therefore excluding primary progressive MS, PPMS and CIS). This most common disease course is characterized by clearly defined attacks of new or increasing neurologic symptoms and, therefore, is less subject to classification bias. Second, only patients with a place of residence available at clinical onset or within the following year were analyzed to avoid migration bias. Third, both previous criteria were applied.

Descriptive statistics were calculated using R software, version 3.2.1 [17]. Spatial scan tests were performed using the SaTScan software program, version 9.4.4 [18].

## Results

### Descriptive data

Over the 2000–2004 period, a total of 848 incident cases of MS were registered in the Brittany region, corresponding to a crude annual incidence rate of 5.8 per 100,000 inhabitants and to an annual standardized incidence rate (using the 2013 European Standard Population) of 5.7 per 100,000 inhabitants. RRMS represented 69.7% (n = 591), CIS 15.2% (n = 129) and PPMS 15.1% (n = 128) of cases. The numbers of incident cases and the breakdown by subtypes were stable across the 5 years of the study period (Table 1).

**Table 1:** Incident cases of multiple sclerosis classified by year of disease onset (Brittany, France, 2000–2004)

	RRMS (n = 591)	CIS (n = 129)	PPMS (n = 128)	Total (n = 848)
*Year of clinical onset*				
2000	124 (69.7%)	24 (13.5%)	30 (16.8%)	178 (100.0%)
2001	115 (71.9%)	22 (13.7%)	23 (14.4%)	160 (100.0%)
2002	117 (72.2%)	25 (15.4%)	20 (12.4%)	162 (100.0%)
2003	119 (67.6%)	31 (17.6%)	26 (14.8%)	176 (100.0%)
2004	116 (67.4%)	27 (15.7%)	29 (16.9%)	172 (100.0%)

*RRMS* relapsing-remitting multiple sclerosis, *CIS* clinically isolated syndrome, *PPMS* primary progressive multiple sclerosis

The mean age of patients at clinical onset was 35.6 (SD = 10.8) years, and 616 (72.6%) were females. The municipality of residence was available within the year following clinical onset for 86% of cases.

### Spatial cluster detection

When considering the whole dataset (n = 848), the spatial scan statistic indicated no statistically significant spatial cluster of MS. More precisely, the most likely cluster of MS consisted of 3 municipalities (radius of 7.2 km, 8040 inhabitants), where 11 cases occurred while 2.61 were expected (*p* value = 0.56). The secondary cluster consisted of 147 municipalities (radius of 49.7 km, 475,901 inhabitants). In this area, 100 cases were observed while 138.00 were expected (*p* value = 0.75).

Sensitivity analyses produced results consistent with the primary analysis, pointing to non-significant clusters of MS incidence (Table 2).

**Table 2:** Characteristics of the two most likely clusters of multiple sclerosis identified in sensitivity analyses (spatial scan statistic, Brittany, France, 2000–2004)

	Radius (km)	Inhabitants	Observed cases	Expected cases	*p* value
RRMS (n = 591)					
Primary cluster	15.6	36,932	0	7.05	0.60
Secondary cluster	10.0	20,857	13	4.34	0.98
Municipality of residence ≤ 1 year after clinical onset (n = 728)					
Primary cluster	30.8	195,187	22	47.68	0.16
Secondary cluster	37.1	288,708	41	69.84	0.49
RRMS and municipality of residence ≤ 1 year after clinical onset (n = 516)					
Primary cluster	16.4	40,516	0	6.86	0.68
Secondary cluster	30.8	195,187	15	33.53	0.71

*RRMS* relapsing-remitting multiple sclerosis

## Discussion

We found little evidence for spatial clustering of MS incidence at the small-area level in either the primary analysis or in the sensitivity analyses, at variance with variations in exposure to putative local environmental factors. In our opinion, these findings are compelling given the features of the study population, the rigorous case ascertainment approach, the fine geographic resolution of the study, and the use of a sound statistical approach for cluster detection based on the spatial scan statistic.

The Brittany region can be considered a natural laboratory for disentangling the influence of potential environmental triggers and the underlying susceptibility of the population. This region has a relatively unique history of population settlement, is relatively isolated, and is considered an ethnically homogeneous population with one of the lowest immigrant rates in France (1.7% in 1999 vs 7.3% at the national level).

The current study is among the few that have evaluated the distribution of incidence rather than prevalence, a much more relevant measure of risk when formulating new etiological hypotheses. Moreover, previous incidence reports were based on cross-sectional ascertainment of cases in which the year of onset was determined by interview at the time of ascertainment and without follow-up to record a potential second event. To circumvent this drawback, a careful population-based and multi-stage process from suspected to confirmed diagnosis was specifically designed to achieve an accurate incidence estimate. The follow-up period stopped 12 years (2016) after the end of the study period (2004), allowing sufficient time to account for changes in the disease course and to assess (even retrospectively) the precise time of the clinical onset of disease. Moreover, we primarily relied on clinical rather than administrative data to identify incident MS cases (and not the reverse, as done in many previous studies). As a result, completeness does not seem to be an issue, as evidenced by the absence of significant low-rate clusters that would have suggested the incomplete ascertainment of cases. Moreover, as all clinical onsets of MS were ascertained by a unique neurologist after medical record review, no spatial bias is to be feared across the study region.

This study benefited from the well-established advantages of the spatial scan statistic. This method adjusts for inhomogeneous population density and resolves the problem of preselection bias by searching for clusters without specifying their size or location. The likelihood ratio-based statistic takes multiple testing into account and delivers a single *p* value. We used municipalities as the basis for our clustering work because their small average size makes them appropriate for revealing local-level inequalities that are often masked when analysis is produced at large area scales. It is good practice to adjust for socioeconomic deprivation, which is known to be an explanatory or a confusion factor of the spatial distribution of various health events. To this end, we introduced a French deprivation index that we have shown to capture both urban and rural deprivation [19].

This study is not without its limitations. First, although 848 cases have been identified among more than 3 million inhabitants during a 5-year period, the possibility of insufficient statistical power to indicate clusters cannot be entirely dismissed. As recommended, we used a large number of replications (9999) to ensure a high statistical power [20]. Unfortunately, just as there is no known analytical way to obtain the *p* values for the scan statistics, there is no analytical way to estimate the statistical power. The only way is to pre-specify alternative hypotheses (through a set of hypothetical cluster locations, sizes and relative risks) and conduct simulations. However, as there is no or little evidence of local environmental risk factors, any alternative hypothesis would be highly questionable. Second, deprivation is measured over geographical areas, assuming that inequalities are partly related to social and environmental influences that exist above the level of the individual. Residual confounding by individual socioeconomic status may, however, still exist. Third, the place of residence was not retrieved at the time of clinical onset for 120 cases (14%), which might have introduced some uncertainty into the spatial analysis if patients moved in the meantime. However, our results are unlikely to suffer from migration bias, as the sensitivity analysis performed on cases for which the municipality of residence was documented within the year following clinical onset was in agreement with the primary analysis, with no cluster being identified. Moreover, residential mobility rates are low in Brittany; in 2002, 79% of migrations occurred within the region, among which 60% occurred between neighboring municipalities with similar characteristics [21]. Fourth, using only the residential address at the time of clinical onset may limit the study’s ability to assess clustering based on past exposures (which may be measured through lifetime residential histories), as a gestational or childhood timing of environmental effects has been suggested [5]. Fifth, our results may be specific to the Brittany region, and caution should be exercised in any attempt to generalize this lack of evidence for local clustering in MS incidence to other geographical areas.

The annual standardized incidence rate here estimated for Brittany (5.7 per 100,000 inhabitants) is close to those reported in French and other European studies based on medical sources [9, 12, 22], keeping in mind that inter-study comparisons are hampered by the marked variability in the methodology, time period and diagnostic criteria [22]. The only study of a similar design (to our best knowledge), reported an onset-adjusted incidence rate of 3.6 per 100,000 inhabitants in the Girona province in Catalonia (Spain) but did not consider PPMS [23].

Our not finding any statistically significant spatial clustering of MS cases in Brittany is in contrast to two recent studies completed in a Canadian province (Manitoba), where significant high-rate and low-rate clusters were identified [6, 7]. However, as a low Gini coefficient was found, the authors concluded that the causes of MS are pervasive and shared across population groups [6]. In French territory, several studies found geographic variations in MS but at much larger geographical scales than the municipality level (*département* or region), highlighting higher prevalence or incidence rates in north-eastern France [8, 24, 25].

With respect to environmental factors, one of the most frequently reported geographical variations is the MS latitude gradient. In a meta-analysis including 650 prevalence estimates from 321 peer-reviewed studies, a significant positive association was found between age-standardized prevalence and latitude [26]. This striking latitudinal gradient in MS prevalence strongly supports a role for environmental factors, which vary with latitude, the most prominent candidates being ultraviolet radiation. In Brittany, we found no evidence of a latitude gradient in MS (which could have taken the form of high-risk clusters on the northern border and/or low-risk clusters on the southern border). If such an effect exists, one can hypothesize that the extent of the study area (approximately 180 km from north to south) is insufficient to reflect the influence of such a large-level risk factor.

## Conclusion

The findings of this study indicate that MS incidence is not markedly affected across space, suggesting that in the years preceding the first clinical expression of the disease, no environmental trigger is operative at the small-area population level.
